# *APOE* Genotype-Function Relationship: Evidence of −491 A/T Promoter Polymorphism Modifying Transcription Control but Not Type 2 Diabetes Risk

**DOI:** 10.1371/journal.pone.0024669

**Published:** 2011-10-18

**Authors:** Hua Geng, Peggy P. Y. Law, Maggie C. Y. Ng, Ting Li, Li-Yun Liang, Tian-Fang Ge, Kam-Bo Wong, Chun Liang, Ronald C. Ma, Wing-Yee So, Juliana C. N. Chan, Yuan-Yuan Ho

**Affiliations:** 1 Department of Biochemistry, The Chinese University of Hong Kong, Shatin, New Territories, Hong Kong SAR, China; 2 Department of Medicine and Therapeutics, Prince of Wales Hospital, The Chinese University of Hong Kong, Shatin, New Territories, Hong Kong SAR, China; 3 Department of Biochemistry, Hong Kong University of Science and Technology, Hong Kong, China; 4 Genetics of Complex Disorder Program, Departments of Biostatistics and Psychiatry, Columbia University, New York, New York, United States of America; The University of Hong Kong, Hong Kong

## Abstract

**Background:**

The apolipoprotein E gene (*APOE*) coding polymorphism modifies the risks of Alzheimer's disease, type 2 diabetes, and coronary heart disease. Aside from the coding variants, single nucleotide polymorphism (SNP) of the *APOE* promoter has also been shown to modify the risk of Alzheimer's disease.

**Methodology/Principal Findings:**

In this study we investigate the genotype-function relationship of *APOE* promoter polymorphism at molecular level and at physiological level: i.e., in transcription control of the gene and in the risk of type 2 diabetes. In molecular studies, the effect of the *APOE* −491A/T (rs449647) polymorphism on gene transcription was accessed by dual-luciferase reporter gene assays. The −491 A to T substitution decreased the activity (p<0.05) of the cloned *APOE* promoter (−1017 to +406). Using the −501 to −481 nucleotide sequence of the *APOE* promoter as a ‘bait’ to screen the human brain cDNA library by yeast one-hybrid system yielded ATF4, an endoplasmic reticulum stress response gene, as one of the interacting factors. Electrophoretic-mobility-shift assays (EMSA) and chromatin immuno-precipitation (ChIP) analyses further substantiated the physical interaction between ATF4 and the *APOE* promoter. Over-expression of ATF4 stimulated *APOE* expression whereas siRNA against ATF4 suppressed the expression of the gene. However, interaction between *APOE* promoter and ATF4 was not −491A/T-specific. At physiological level, the genotype-function relationship of *APOE* promoter polymorphism was studied in type 2 diabetes. In 630 cases and 595 controls, three *APOE* promoter SNPs −491A/T, −219G/T (rs405509), and +113G/C (rs440446) were genotyped and tested for association with type 2 diabetes in Hong Kong Chinese. No SNP or haplotype association with type 2 diabetes was detected.

**Conclusions/Significance:**

At molecular level, polymorphism −491A/T and ATF4 elicit independent control of *APOE* gene expression. At physiological level, no genotype-risk association was detected between the studied *APOE* promoter SNPs and type 2 diabetes in Hong Kong Chinese.

## Introduction

Type 2 diabetes is a multi-factorial and polygenic disease which makes up 90% of all cases of diabetes. Dyslipidemia is one of the risk factors for type 2 diabetes as well as for diabetic complications, such as coronary heart disease, diabetic nephropathy and retinopathy [1], [2].

Apolipoprotein E (apoE) is a 34 kD protein which plays a central role in lipid metabolism. Two coding polymorphisms of the gene resulting in three protein variants apoE2, apoE3 and apoE4 incur isoform-dependent risk associations with Alzheimer's disease, atherosclerosis and coronary heart disease [3]. ApoE is also an important molecule in the development and progression of diabetes. A recent meta-analysis of genome-wide linkage studies of quantitative lipid traits in families ascertained for type 2 diabetes with diverse ethnic backgrounds identified one of the linkage region for lipid traits on chromosome 19q13.13-13.43 which included the *APOE* gene locus (19q13.2) [4]. Another meta-analysis on data of 5423 cases and 8197 controls extracted from 30 studies provided evidence that the *APOE2* allele carriers have elevated risk for type 2 diabetes [5]. Aside from the isoform-dependent effects, plasma apoE has been associated with the risk of cardiovascular diseases in a dose-dependent manner [6]. An increment of plasma apoE in type 2 diabetic patients as compared to healthy controls has been reported [7]. It is conceivable that the transcriptional activity of *APOE* may affect plasma concentration of the protein. An increasing body of evidence has associated *APOE* promoter polymorphisms with human diseases. For example, the *APOE* promoter −491A genotype has been associated with a higher plasma level of apoE and increased risk for Alzheimer's disease as compared to its −491T counterpart [8], [9]. In spite of the association between *APOE* promoter polymorphisms and disease risks, the underlying mechanisms responsible for controlling *APOE* gene expression remain elusive.

In this study, we aimed at elucidating the genotype-function relationship of *APOE* promoter polymorphism at molecular and physiological levels. At molecular level, we further investigated the transcriptional control mechanism at the −491A/T-spanning region of *APOE*. At physiological level, we examined the association of *APOE* promoter polymorphisms −491A/T (rs449647), −219G/T (rs405509) and +113G/C (rs440446) with the risk of type 2 diabetes. These three SNPs were chosen for analysis based on their previously reported association with Alzheimer's disease and coronary heart disease [10], [11]. Investigation of association between these SNPs and type 2 diabetes has not been reported. Our molecular studies demonstrate for the first time that ATF4, a key transcription factor mediating ER (endoplasmic reticulum) stress response and regulates lipid and glucose homeostasis in mammals [12], is interactive with the *APOE* promoter and controls the expression of the gene independent of the control elicited by the −491A/T polymorphism. At physiological level, no association was detected between the three *APOE* promoter SNPs and the risk of type 2 diabetes in Hong Kong Chinese.

## Results

### −491A/T polymorphism regulates *APOE* promoter activity

**Figure 1:**
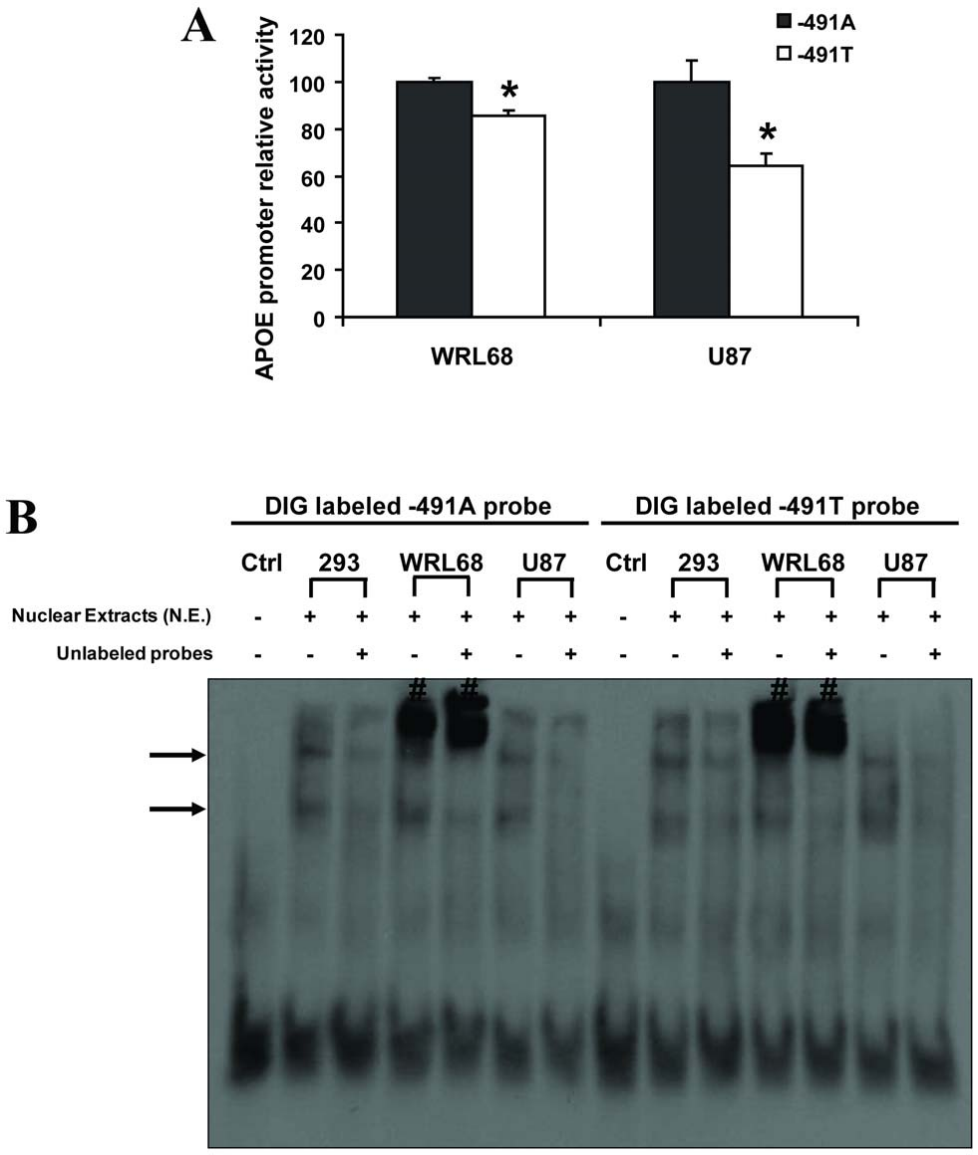
*APOE* promoter −491A/T polymorphism is a functional variant. (A) −491A to T single nucleotide substitution significantly decreases the transcriptional activity of the *APOE* promoter. *APOE* promoter firefly luciferase reporter constructs (−1017 to +406) containing A or T at −491 site are designated −491A or −491T, respectively. Data are presented as *APOE* promoter (−491T) activity relative to that of its −491A counterpart (value set at 100%). Data are obtained from three to five independent experiments in triplicate set up. * *p*<0.05 by Student's *t*-test. (B) Interaction between nuclear factors and *APOE* promoter −491A/T-spanning sequence. EMSA assays were performed in 20 µl reaction system containing 3 nM of DIG-labeled −491A or −491T probe (−521 to −461)±5 µg of cell nuclear extracts. The absence (−) or the presence (+) of 192 nM of unlabeled corresponding −491A or −491T probes served as the control or competition conditions for specific binding between the DIG-labeled probe and nuclear proteins. Shift-bands are indicated by arrowheads. Non-specific shift-bands are indicated by “#”. WRL68: human hepatic embryonic cell line; U87: human astrocytic cell line; 293: human kidney epithelial cell line.

The effects of −491A/T polymorphism on the activities of the *APOE* gene were analyzed by dual-luciferase reporter assay. Figure 1A shows that −491A to T single nucleotide substitution significantly changed *APOE* promoter activity in WRL-68 (human hepatic embryonic) and U-87 (human astrocytic) cell lines (decrease of 14% and 36%, respectively, *p*<0.05). These results support that the *APOE* promoter −491 polymorphism is functionally active and elicits similar regulatory effects on *APOE* transcription in human cell lines of liver and brain origins. Further analyses by EMSA revealed the interaction of nuclear proteins with *APOE* promoter −491A/T-spanning sequence (−521 to −461) (Figure 1B). Subsequent studies were carried out to identify the potential interacting transcription factors with *APOE* promoter −491A/T-spanning region.

### ATF4 is a candidate transcription factor binding to *APOE* promoter −491A/T- spanning sequence

Yeast one-hybrid screening of a human brain cDNA library identified ATF4 being one of the candidate transcription factors interactive with the *APOE* −491-spanning sequence. ATF4 belongs to ATF/CREB transcription factor family that mainly involves in the PERK endoplasmic reticulum (ER) stress response. It is well known that the dysfunction of ER stress responses can result in various diseases including diabetes, Alzheimer's disease and inflammation [13]. Further bioinformatic analysis using the TRANSFAC 6.0 database identified a sequence homologous to the ATF/CRE core binding site (TGACCTTA, −486 to −479) adjacent to the studied *APOE* promoter −491A/T-spanning region. Taken together, ATF4 was selected as a candidate transcription factor for further investigation of its interaction with *APOE* promoter and the regulation of *APOE* gene transcription.

### ATF4 interacts with *APOE* promoter −491A/T-spanning sequence *in vitro* and *in vivo*

**Figure 2:**
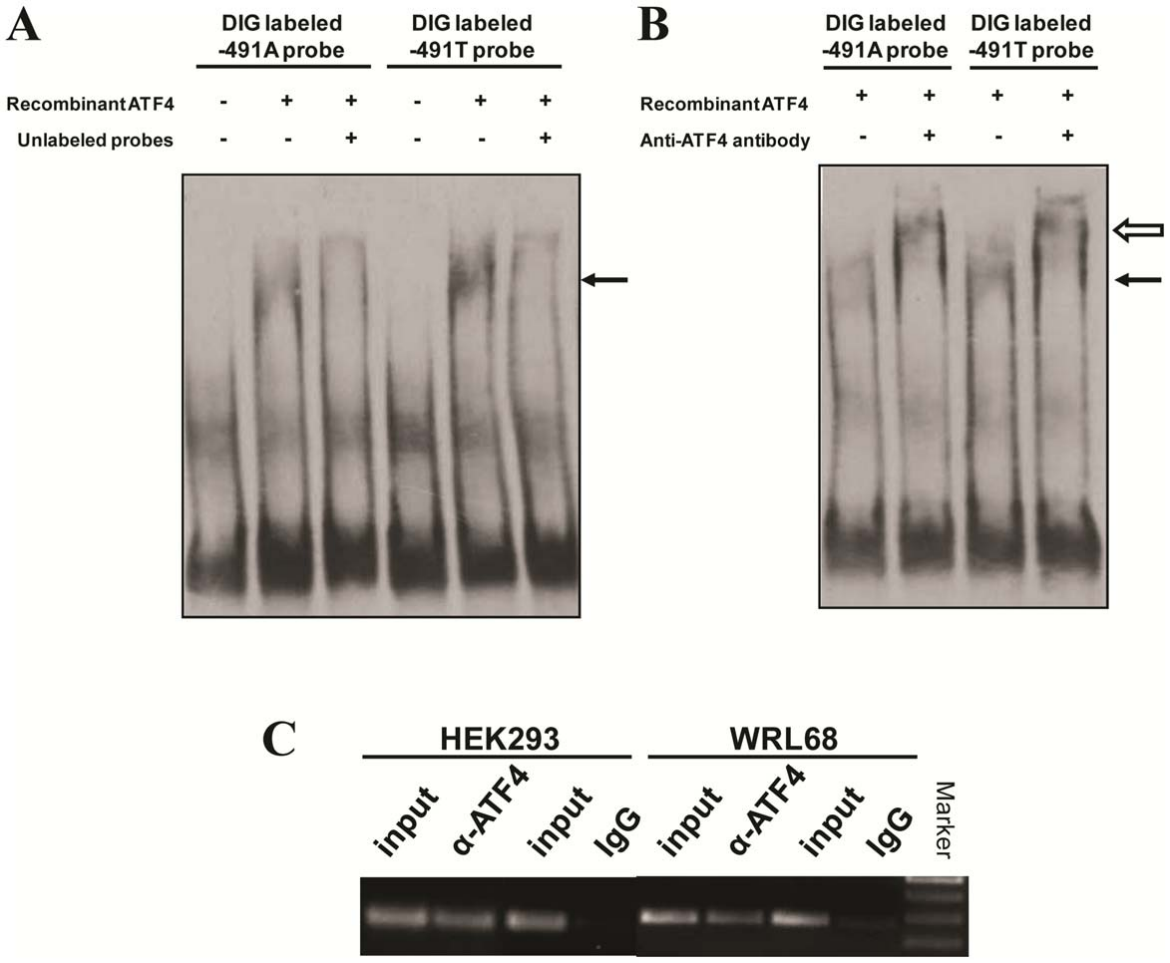
ATF4 physically interacts with *APOE* promoter −491-spanning region. (A) EMSA analyses were performed in 20 µl reaction system containing 3 nM of DIG-labeled −491A or −491T probe (−521 to −461)±1.2 µM of the purified His-tagged ATF4 protein, the shift-bands are indicated by solid arrowhead. (B) Super-shift assays are performed by the addition of ATF4 antibody into reaction mixture containing −491A or −491T probes and 0.7 µM purified ATF4 protein. The super-shift bands are indicated by unfilled arrowhead. (C) Chromatin Immunoprecipitation (ChIP) assays confirmed the *in vivo* interaction of *APOE* promoter −491-spanning sequence and ATF4 in 293 (−491AA genotype) and WRL68 (−491TT genotype) cells.

To further verify the direct interaction between *APOE* promoter and ATF4, EMSA was performed using purified recombinant His-tagged ATF4 and −491A or −491T probes (−521 to −461). Clear shift of bands were detected for both −491A and T probes (Figure 2A). Addition of excessive unlabeled probes completely competed with the labeled probe for ATF4 binding, indicating this binding is specific. Further super-shift assay with ATF4-specific antibody lead to a super-shift band for both −491A and −491T probes as shown in Figure 2B. ChIP assays were performed to further confirm the binding of ATF4 and *APOE* promoter −491A/T-spanning locus *in vivo*. Results in Figure 2C showed that *APOE* promoter −617 to −344 region encompassing the −491A/T-spanning site could be amplified only from the anti-ATF4 antibody immuno-precipitated complex, while PCR product was merely detectable in the IgG control both in 293 cells (−491AA genotype) and WRL-68 cells (−491TT genotype). Taken together, these results further substantiated the physical interaction between ATF4 and the *APOE* promoter at the −491A/T-spanning region both *in vitro* and *in vivo*.

### ATF4 regulates *APOE* transcription and expression

**Figure 3:**
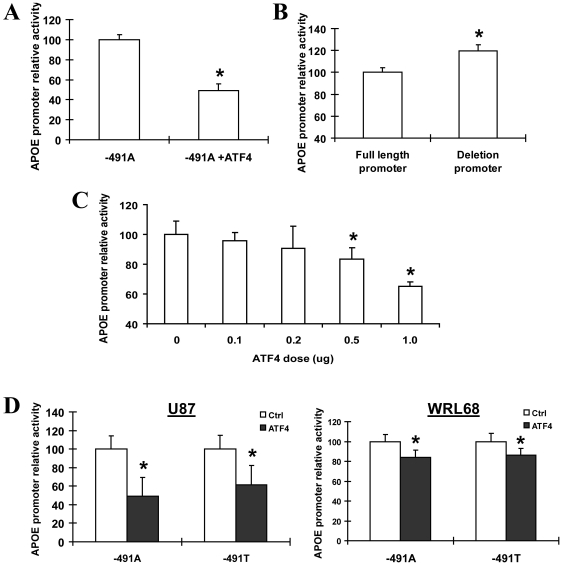
ATF4 regulates cloned *APOE* promoter activity. (A) The pcDNA3.1-ATF4 or pcDNA3.1 control vector was transfected into U-87 cells together with *APOE* promoter luciferase reporters and internal control plasmid Renilla, the *APOE* promoter activity was examined by dual-luciferase assay. (B) *APOE* promoter reporter (with −491A) or the deletion of the putative ATF4 binding sequence (−487 to −469) of cloned *APOE* promoter (−1017 to +406 with −491A) was transfected into U-87 cells, luciferase assay was then performed to determine the activity of the promoter. (C) A series of 0.1 ug to 1.0 ug of ATF4 plasmids were transfected into WRL-68 cells together with *APOE* promoter reporters to determine the dose-dependent effect of ATF4 on regulating *APOE* promoter activity. (D) ATF4 over-expression significantly down-regulated the activities of cloned *APOE* promoters both with −491A and −491T allelic forms in U-87 and WRL-68 cell lines by dual-luciferase assay. The Y-axes represent the percent activity of the *APOE* promoter relative to the control condition (set at 100%). All data were collected from three independent experiments in triplicate set up, *p<0.05.

To elucidate the biological effects of ATF4 interaction with *APOE* promoter −491A/T-spanning region, the *APOE* promoter firefly reporter constructs (with −491A or −491T allelic form), pcDNA3.1-ATF4 mammalian expression vector (or pcDNA3.1 empty vector) and the internal control Renilla luciferase reporter vector were co-transfected into mammalian cells. Dual-luciferase reporter assays showed that ATF4 over-expression significantly suppressed the *APOE* promoter (with −491A) activity in U-87 cells by about 50% (Figure 3A). Site-directed mutagenesis was performed to generate an *APOE* promoter deletion mutant Δ(−487 to −469) reporter construct spanning the putative ATF4 binding site. Abolishing this putative ATF4 binding site resulted in 20% increase of *APOE* promoter activity as compared to the full-length *APOE* promoter (−491A allelic form) (Figure 3B). Furthermore, the suppressive effect of ATF4 on *APOE* promoter activity was dose-dependent with statistical significant effects observed at higher dosages (Figure 3C). ATF4 over-expression significantly down-regulated the activities of cloned *APOE* promoters both in −491A and −491T allelic forms in U-87 and WRL-68 cells (Figure 3D) although no apparent allelic-difference was observed. These results strongly support that ATF4 modulates the transcriptional activity of *APOE* promoter.

**Figure 4:**
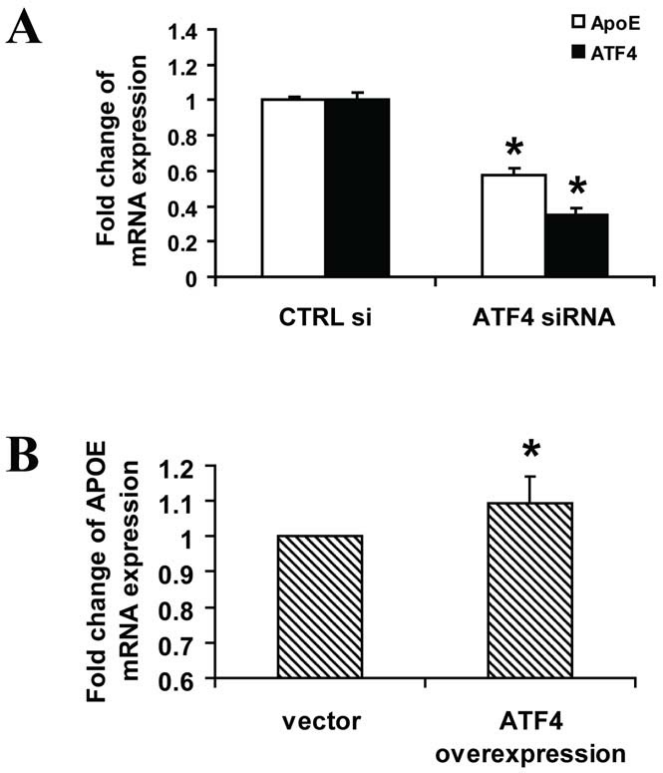
ATF4 modulates endogenous *APOE* expression. (A) WRL-68 cells were transfected with 100 pmol of ATF4 siRNA or control siRNA. The expression levels of *APOE* and ATF4 were measured by Q-PCR after normalizing to beta-actin. Q-PCR data of cells treated with control siRNA were set as 1. (B) WRL-68 cells were transfected with 1 µg of pcDNA3.1-ATF4 mammalian expression vector (or pcDNA3.1 empty vector). Results are presented as the fold change of endogenous *APOE* mRNA expression relative to that of the cells transfected with pcDNA3.1 vector controls (value set at 1). Data shown are from 3–4 independent experiments in triplicate set-up, *p<0.05.

We next examined the effects of ATF4 on the expression of endogenous *APOE*. Blockage of the endogenous ATF4 expression by siRNA in WRL68 human hepatic cells caused a 42% reduction of endogenous *APOE* expression (Figure 4A). On the other hand, over-expression of ATF4 enhanced *APOE* mRNA expression (Figure 4B). These results further substantiated the functional role of ATF4 in regulating *APOE* gene expression.

### No association was detected between *APOE* promoter polymorphism and the risk of type 2 diabetes

To investigate the genotype-function relationship of *APOE* promoter polymorphism at physiological level, we tested the association between *APOE* promoter SNPs and the risk of type 2 diabetes in Hong Kong Chinese. In our case-control study, the control subjects (41.37±10.48 years) were slightly older than the type 2 diabetic patients (40.07±8.39 years) but such difference is not statistically significant after adjustment of multiple testing. There is a female preponderance in the type 2 diabetes cohort (55.1% in controls and 61% in type 2 diabetes, p = 0.039). Type 2 diabetic patients had significantly higher BMI, WHR, blood pressure, more adverse lipid profiles (high total cholesterol, high LDL-C, low HDL-c and high TG), and higher plasma glucose levels than non-diabetic control subjects (p<0.001) (Table S1).

All three *APOE* proximal promoter polymorphisms were successfully genotyped with high call rates: 95.9% for −491A/T (rs449647), 99.8% for −219G/T (rs405509) and 98.8% for +113G/C (rs440446). All genotype distributions were in Hardy-Weinberg equilibrium (HWE) in both type 2 diabetes and non-diabetic control groups. *APOE* promoter −491A/T, −219G/T and +113G/C genotype distributions and allele frequencies were similar between the control and type 2 diabetes groups (Table 1). Analysis with gender stratification did not reveal an association between these SNPs and type 2 diabetes either. Linkage disequilibrium (LD) analysis indicated that −219G/T and +113G/C polymorphisms form an LD block which is not linked to the *APOE* −491A/T polymorphism (Table S2). No association was detected between haplotypes (formed between −491A/T and the −219G/T-tagged LD block) and type 2 diabetes.

**Table 1:** Associations between *APOE* polymorphisms and the risk of type 2 diabetes.

	Genotype number (percentage)				HWE	Allele number (percentage)		
−491A/T	N	AA	AT	TT		N	A	T
Non-diabetic controls	589	546 (92.7%)	43 (7.3%)	0 (0)	0.358	1178	1135 (96.3%)	43 (3.7%)
Type 2 diabetes	586	544 (92.8%)	42 (7.2%)	0 (0)	0.368	1172	1130 (96.4%)	42 (3.6%)
P value		0.929					A: 0.933	
OR (95% CI)		1.02 (0.67–1.59)^a^					1.02 (0.66–1.57)	
−219G/T	N	GG	GT	TT		N	G	T
Non-diabetic controls	594	78 (13.1%)	288 (48.5%)	228 (38.4%)	0.384	1188	444 (37.4%)	744 (62.6%)
Type 2 diabetes	629	84 (13.4%)	291 (46.3%)	254 (40.1%)	0.964	1258	459 (36.5%)	799 (63.5%)
P value	2×3: 0.727		T+: 0.909^b^	G+: 0.475^c^			G: 0.649	
OR (95% CI)			0.98 (0.70–1.37)^b^	0.92 (0.73–1.16)^c^			0.96 (0.82–1.14)	
+113G/C	N	GG	GC	CC		N	G	C
Non-diabetic controls	586	126 (21.5%)	291 (49.7%%)	169 (28.8%)	0.972	1172	543 (46.3%)	629 (53.7%)
Type 2 diabetes	624	126 (20.2%)	306 (49.0%)	192 (30.8%)	0.839	1248	558 (44.7%)	690 (55.3%)
P value	2×3: 0.722		C+: 0.575^d^	G+: 0.463^e^			G: 0.424	
OR (95% CI)			1.08 (0.82–1.43)^d^	0.91 (0.71–1.17)^e^			0.94 (0.80–1.10)	

HWE: *p* value for Hardy-Weinberg equilibrium; *p* values and ORs of genotypes were calculated by comparison between type 2 diabetes and non-diabetic control groups: AA vs. AT ^a^, T+ vs. GG ^b^, TT vs. G+ ^c^, C+ vs. GG ^d^, CC vs. G+ ^e^.

## Discussion

The results from the current and previous studies indicated that the polymorphism of *APOE* promoter at the −491 site is functionally significant in modifying *APOE* gene transcription as well as in the development of diseases [6], [8]. However, the transcriptional control mechanism of *APOE* at this locus has not been well characterized. In this study, we presented evidence that transcription factor ATF4 functionally regulates and physically interacts with the *APOE* promoter. Such regulation is independent of the −491A/T polymorphism.

ATF4 is widely expressed in different organs including liver, kidney, brain, spleen, heart, thymus, lung, blood cells and fibroblasts [14]. It belongs to the ATF/CREB bZIP transcription factor family and is a key transcription factor controlled under the PERK signaling pathway which is up-regulated by ER stress – a process referring to the excessive cellular protein load relative to the reserve of ER chaperones required for correct folding of newly synthesized membrane or secretary proteins [15]. ATF4 translation is induced upon ER stress followed by its migration to nucleus to induce antioxidant genes and genes of the ER protein maturation machinery [16].

ER stress and *APOE* have been independently associated with neurodegenerative diseases and atherosclerosis [6], [8], [9], [13]. Also ER stress is related to the increased ATF4 expression as well as β-cell apoptosis and the subsequent development of diabetes [17], [18]. Given the important role of ATF4 in ER stress responses, the control of *APOE* expression by ATF4 demonstrated in this study provides a plausible link between ER stress and *APOE*-mediated cellular function in disease processes.

Previous studies have reported the difference of *APOE* promoter transcription activities elicited by the −491A/T polymorphism in human hepatocellular carcinoma HepG2 cells [19]. We have obtained similar results in other cell types representing the major apoE production tissues including WRL-68 human embryonic hepatocytes and U-87 human astrocytes. *APOE* promoter in the −491A allelic form was associated with higher transcriptional activity as compared to its −491T counterpart in both cell lines. The magnitude of change was greater in astrocytes than that of the liver cells. This may be explained by tissue-specific transcription activities. These observations support that *APOE* promoter −491A/T polymorphism is functionally active in regulating *APOE* promoter in different tissues and justified further characterization of the transcriptional control mechanism within this region.

It has been shown that nuclear proteins from HepG2 (hepatoma) and NB (neuroblastoma) cells can bind to the −491-spanning region of the *APOE* promoter [20], [21] . However, there has been no further resolution of the interacting proteins with the *APOE* −491-spanning sequence. In this study, yeast one-hybrid screening and EMSA analyses identified and verified the physical interaction between *APOE* promoter −491–spanning sequence and the transcription factor ATF4. Using the purified recombinant ATF4 protein and the −491A/T allelic oligonucleotide probes, we found ATF4 interactive with these probes *in vitro*. The results of ChIP assays further supported such physical interaction *in vivo*.

The dual-luciferase assays reported the functional effects of ATF4 on *APOE* promoter. ATF4 can suppress the activity of *APOE* promoter in WRL-68 and U-87 cells. This suppressive effect is common to both −491A and T allelic forms. Consistently, the suppressive effect of ATF4 did not significantly differ in magnitude for the two −491 allelic forms of the promoter in both cell lines examined.

It is curious that the cloned *APOE* promoter and endogenous *APOE* expression showed opposite responses to ATF4 over-expression. It is well known that ATF4 can partner with different transcription factors to act as a transcription repressor or activator [22]. It is likely that the endogenous promoter can recruit a wider spectrum of ATF4 partners as compared to the cloned promoter and thus renders such difference in response. Alternatively, the endogenous *APOE* promoter may recruit repressors which bind outside of the sequence of the cloned promoter to elicit a different response.

The −491A/T-dependent difference in *APOE* promoter activity observed in this study and previous report cannot be explained by ATF4 alone. It is speculated that other transcription factors are involved in the −491A/T-dependent transcription regulation of *APOE*. Such speculation is supported by two lines of evidence. First, the EMSA assays using mammalian cell nuclear extracts showed two shift-bands, indicating more than one group of nuclear proteins can bind to the *APOE* promoter −491-spanning sequence. Second, ATF4 is known to interact with several transcription factors, i.e., C/EBP and p300 which have putative binding sites within the studied *APOE* promoter −491-spanning sequence (predicted by TRANSFAC 6.0 database) [23], [24], [25]. It is likely that C/EBP, p300 as well as other putative factors interactive with ATF4 can elicit general and/or −491A/T-specific control to *APOE* transcription. Further investigation is required to elucidate the identities and interactions of additional transcription factors with the −491-spanning region of *APOE* promoter.

At molecular level, we have demonstrated the relationship between the *APOE* −491A/T genotype and the transcription phenotype of the gene. At physiological level, the association between the *APOE* promoter polymorphism and the risk of type 2 diabetes was tested for the −491A/T, as well as for two additional SNPs −291G/T and +113G/C. The fact that the −291 and +113 loci belong to a different LD block adjacent to the −491 locus opened the possibility for further exploring the *APOE* promoter genotype-function relationship by haplotype analysis. No SNP or haplotype association was detected between the *APOE* promoter and the risk of type 2 diabetes. Since we previously reported an association between *APOM* gene polymorphism and the disease duration of type 2 diabetes [26], such association was also tested for the three *APOE* promoter SNPs. Again, we did not detect an association between *APOE* promoter SNPs and the duration of type 2 diabetes (data not shown). However, it is important to note that with the given effect and sample size, our study is underpowered to reject the null hypothesis.

In conclusion, at molecular level, *APOE* gene transcription is under the independent control of *the* promoter −491A/T polymorphism and the ER stress- responsive transcription factor, ATF4. At physiological level, there is a lack of evidence of association between the three *APOE* promoter SNPs −491A/T (rs449647), −219G/T (rs405509), and +113G/C (rs440446) and the risk of type 2 diabetes. These results encourage further investigation of *APOE* promoter regulation under ER stress but discourage the application of the three studied SNPs as risk markers for type 2 diabetes.

## Materials and Methods

### Cell culture

293 (human kidney epithelial), WRL-68 (human hepatic embryonic), and U-87 (human astrocytic) cell lines were obtained from American Type Culture Collection (Manassas, VA, USA). Cells were maintained in high glucose DMEM or RPMI 1640 (Gibco, Carlsbad, CA, USA) containing sodium bicarbonate, 10% FBS, 100 unit/mL penicillin and 100 µg/mL streptomycin at 37°C in 5% CO_2_.

### Yeast one-hybrid screening

Yeast one-hybrid system was adopted to screen for candidate transcription factors interacting with the *APOE* promoter −491A/T-spanning sequence. Yeast strain *Saccharomyces cerevisiae* YM4271 and reporter vector pHISi-1 carrying the *HIS3* reporter gene were obtained from BD Bioscience (Palo Alto, CA, USA). The reporter constructs pHISi-1-491A and pHISi-1-491T were generated with three head-to-tail copies of the 21-bp bait sequence (5′-CTG GTC TCA AAC TCC TGA CCT-3′) or (5′-CTG GTC TCA ATC TCC TGA CCT-3′), which corresponds to the human *APOE* promoter −491A/T-spanning sequence (−501 to −481).

Two independent sets of screenings for the GAL4 activating domain AD tagged human brain MATCHMAKER cDNA library (BD Bioscience) were performed with the constructed yeast reporter strains, pHISi-1-491A and pHISi-1-491T, respectively. Following the BD Clontech MATCHMAKER one-hybrid system standard procedure, after three rounds of selections, 25 and 23 positive clones were recovered from pHISi-1-491A and pHISi-1-491T baits screening respectively, with DNA sequencing performed to confirm the identity of the recovered clones (service provided by Macrogen, Seoul, Korea).

### Plasmid constructs

Previously characterized *APOE* proximal promoter region −1017 to +406 containing the −491A allelic form (GenBank accession No.: *AF055343*) [19] was amplified by PCR using commercial human genomic DNA as template (Promega, Madison, WI, USA) and subcloned into firefly luciferase reporter pGL3-basic vector (Promega). Site-directed mutagenesis was performed using the QuikChange Site-Directed Mutagenesis Kit (Stratagene, La Jolla, CA, USA) to introduce A to T substitution at position −491. *APOE* promoter −491A/T-spanning sequence deletion mutant Δ (−487 to −469) was generated by PCR overlap extension mutagenesis method.

ATF4 open reading frame (GenBank accession No.: *BC073990*) was obtained by RT-PCR using 293 cells and subcloned into both mammalian expression vector pcDNA3.1(+) (Invitrogen, California, USA) and *E. coli* expression vector pET507a, an in-house modified vector from the pET3d vector (Novagen, Darmstadt, Germany).

### Cell transfection

For ATF4 overexpression, 1 µg of pcDNA3.1-ATF4 expression vector or the control pcDNA3.1 vector was transiently transfected into the cells using Lipofectamine2000 (Invitrogen) method following manufacturer's instructions. Cells were harvested for RNA extraction 30 hrs post-transfection.

For ATF4 gene silencing, cells were transfected with 100 pmol of ATF4 siRNA (5′-gcc uag guc ucu uag aug att-3′ and 5′-uca ucu aag aga ccu agg ctt-3′) or an siRNA control which has a sequence corresponding to ATF4 siRNA but mutated on five nucleotides (5′-gcg uag uuc gcu aag gug att-3′ and 5′-uca ccu uag cga acu acg ctt-3′) by Lipofectamine 2000 reagent (Invitrogen). Medium was refreshed 4 h after transfection. The cells were harvested for RNA extraction 2 days later.

### RNA extraction and real-time PCR

Total RNA was extracted from the cells using Tri Reagent (Molecular Research Center, Inc, Cincinnati, OH, USA). Reverse transcription (RT) was performed using 5 µg of total RNA in a total reaction volume of 20 µl by MMLV reverse transcriptase system (GE Healthcare, Buckinghamshire, UK).

Real-time quantitative PCR was performed on the ABI Prism 7500 Fast Real-Time PCR System (Applied Biosystems, Foster City, CA, USA). The TaqMan probe and primer assays used were Hs00171168_m1 (ABI) for ApoE and Hs00909569_g1 (ABI) for ATF4, respectively. The relative mRNA levels were estimated by the standard method using beta-actin as the reference gene.

### Purification of recombinant ATF4 in *E.coli*

The pET507a-ATF4 expression plasmids were transformed into *E.coli BL21* (DE3, pLysS) strain (Novagen) in LB medium containing the appropriate antibiotics (50 µg/ml of chloramphenicol and 100 µg/ml of ampicillin). The expression of His-tagged ATF4 protein was induced by 0.2 mM of isopropyl-β-thiogalactopyranose and harvested after 5 hrs culture at 37°C. The HiTrap™ Chelating HP column (GE Healthcare, Amersham, UK) pre-charged with 0.1 M of NiSO_4_ was used for purification of His-tagged ATF4 following the standard method [27].

### Cell nuclear extract preparation

Cells nuclear extracts were prepared by NucBuster™ Protein Extraction kit (Novagen). The protein concentrations of nuclear extracts were determined by DC protein assay kit (Bio-Rad, Hercules, CA, USA) using BSA as a standard.

### Electrophoretic mobility shift assay (EMSA)

The EMSA assays were performed with the use of mammalian cell nuclear extracts or purified ATF4 and DIG-labeled oligonucleotide probes according to the manufacturer's instructions (Roche, Penzberg, Germany). The 61-bp *APOE* promoter −491A/T-spanning sequence were used as the double-stranded probes: −491A 5′-GTT TCA CCA TGT TGG CCA GGC TGG TCT CAA ACT CCT GAC CTT AAG TGA TTC GCC CAC TGT G-3′ and -491T 5′-GTT TCA CCA TGT TGG CCA GGC TGG TCT CAA TCT CCT GAC CTT AAG TGA TTC GCC CAC TGT G-3′. For the supershift assays, anti-ATF4 antibody (Santa Cruz, CA, USA) was used.

### Chromatin immunoprecipitation (ChIP) assay

A total of 5×10^7^ cells were used for each ChIP assay and immunoprecipitated with 1 µg of ATF4 antibody following the standard protocol [28]. The immunoprecipitated genomic DNA was then subjected to PCR using the following primers adjacent to the *APOE* promoter −491A/T-spanning sequences: forward primer 5′-TTC AAG CGA TTC TCC TGC CT-3′; reverse primer 5′-TGG GGA TCT GGA CTC CTG GA-3′.

### Dual-luciferase reporter assay

*APOE* promoter firefly luciferase reporter constructs were transfected into cells by Lipofectamine™ 2000 reagent (Invitrogen) together with Renilla luciferase reporter pRL-CMV vector (Promega) which provided the internal control for transfection. To evaluate the effect of ATF4 on *APOE* promoter activity, *APOE* promoter firefly luciferase reporter constructs and pcDNA3.1-ATF4 expression construct (or pcDNA3.1 empty vector), together with the Renilla luciferase reporter vector were co-transfected into cells by Lipofectamine™ 2000 reagent. Cells were harvested 30 hrs post-transfection and luciferase activities were analyzed by the dual-luciferase assay kit (Promega). The luminometric measurements were performed with a Lumat LB9501 luminometer (Berthold Technologies, Wildbad, Germany).

### Case selection

All subjects were of southern Han Chinese ancestry residing in Hong Kong. The population consisted of 630 unrelated early-onset (diagnosed at ≤40 years) type 2 diabetic patients selected from Hong Kong Diabetes Registry [29] and 595 non-diabetic control subjects [fasting plasma glucose (FPG)<6.1 mmol/l] recruited from the general population participating in a community-based cardiovascular risk screening program as well as hospital staff. The use of the early-onset patients aimed at enriching the genetic background. The clinical characteristics of the study population are summarized in Table S1. This study was approved by the Clinical Research Ethics Committee of the Chinese University of Hong Kong. An informed consent was obtained for each participant. All subjects underwent detailed clinical investigation as described previously [29], [30]. For each participant, a fasting blood sample was collected for the measurement of plasma glucose, lipid profile and for extraction of genomic DNA.

### Genotyping of *APOE* promoter polymorphisms

DNA samples were extracted from whole blood using the phenol-chloroform method. *APOE* proximal promoter −491A/T (rs449647), −219G/T (rs405509) and +113G/C (rs440446) genotypes were determined by Sequenom analysis (service provided by McGill University and Genome Quebec Innovation Centre, Montreal, Canada).

### Statistics

In the case-control study, *APOE* promoter SNPs genotypes (grouped into 2×3 table) were compared by chi-square test. Hardy-Weinberg equilibrium (HWE) was assessed by chi-square test in each study group separately. Odds ratios (OR) with 95% confidence interval were calculated by Epi6 software (World Health Organization, Geneva, Switzerland). Intragenic linkage disequilibrium (LD) in pairs of SNPs and haplotype imputation were analyzed by Haploview (Broad Institute of MIT and Harvard, USA, version 4.2). For the dual-luciferase reporter assays, results were analyzed by Student's t-test or ANOVA with statistical significance threshold set at p<0.05. Statistical analyses were performed using the SPSS program (SPSS version 12.0, Chicago, IL, USA).

## Supporting Information

Table S1 *P* value: calculated by comparing Non-diabetic control vs. Type 2 diabetes groups.
(DOC)

Table S2 D′>80 indicates the existence of linkage disequilibrium between the two markers. r^2^ is related to the power of LD mapping in association studies. When D′>80 and r^2^ is close to 1, the two markers are in LD and most probably display similar disease association profile.
(DOC)
