# Supplementary material for: APOE Genotype-Function Relationship: Evidence of −491 A/T Promoter Polymorphism Modifying Transcription Control but Not Type 2 Diabetes Risk
Source: PLoS One. 2011 Oct 18;6(10):e24669. doi: 10.1371/journal.pone.0024669 (PMC3196492; doi:10.1371/journal.pone.0024669)
Supplement: Table S1 — P value: calculated by comparing Non-diabetic control vs. Type 2 diabetes groups. (DOC) [file pone.0024669.s001.doc]

**Table S1. Correlations between clinical parameters and type 2 diabetes (Non-diabetic controls vs. type 2 diabetes).**

|  | Non-diabetic control | Type 2 diabetes | *P* value |
| --- | --- | --- | --- |
| Number of subjects | 595 | 630 | - |
| Sex (% men) | 44.9% | 39.0% | 0.039 |
| Age (years) | 41.37 ± 10.48 | 40.07 ± 8.39 | 0.016 |
| BMI (kg/m^2^) | 22.9 ± 3.30 | 25.71 ± 4.60 | <0.001 |
| Waist-hip ratio | 0.82 ± 0.07 | 0.86 ± 0.07 | <0.001 |
| Men | 0.87 ± 0.06 | 0.89 ± 0.06 | <0.001 |
| Women | 0.78 ± 0.06 | 0.84 ± 0.07 | <0.001 |
| Systolic blood pressure (mmHg) | 115.23 ± 16.43 | 125.12 ± 18.03 | <0.001 |
| Diastolic blood pressure (mmHg) | 72.18 ± 11.28 | 75.29 ± 10.37 | <0.001 |
| Fasting plasma glucose (mM) | 4.83 ± 0.41 | 8.95 ± 3.38 | <0.001 |
| Total cholesterol (mM) | 5.03 ± 0.95 | 5.30 ± 1.16 | <0.001 |
| Plasma creatinine (μM) | 76.9 ± 17.20 | 71.71 ± 27.5 | <0.001 |
| HDL-C (mM) | 1.56 ± 0.43 | 1.25 ± 0.37 | <0.001 |
| LDL-C (mM) | 3.00 ± 0.83 | 3.28 ± 0.92 | <0.001 |
| Triglyceride (mM) | 1.10 ± 0.79 | 1.84 ± 2.13 | <0.001 |
