# Supplementary material for: APOE Genotype-Function Relationship: Evidence of −491 A/T Promoter Polymorphism Modifying Transcription Control but Not Type 2 Diabetes Risk
Source: PLoS One. 2011 Oct 18;6(10):e24669. doi: 10.1371/journal.pone.0024669 (PMC3196492; doi:10.1371/journal.pone.0024669)
Supplement: Table S2 — D′>80 indicates the existence of linkage disequilibrium between the two markers. r2 is related to the power of LD mapping in association studies. When D′>80 and r2 is close to 1, the two markers are in LD and most probably display similar disease association profile. (DOC) [file pone.0024669.s002.doc]

**Table S2. Linkage disequilibrium (LD) analysis of *APOE* promoter polymorphisms**

| Marker-1 | Marker-2 | r^2^ | D' |
| --- | --- | --- | --- |
| rs449647 | rs405509 | 0.01 | 53 |
| rs449647 | rs440446 | 0.02 | 73 |
| rs405509 | rs440446 | 0.66 | 98 |
